# Interleukin‐10 Modified Human Mesenchymal Stromal Cells Markedly Alleviated Periodontitis by Inducing Macrophage M2 Polarisation

**DOI:** 10.1111/jcmm.70934

**Published:** 2025-11-03

**Authors:** Aerali Heinayati, Xiaowei Jiang, Tianyun Gao, Liudi Wang, Bin Wang, Haiyan Qin

**Affiliations:** ^1^ Department of Stomatology, Nanjing Drum Tower Hospital The Affiliated Hospital of Nanjing University Medical School Nanjing China; ^2^ Department of Stomatology, Fuyang People's Hospital Anhui Medical University Fuyang China; ^3^ Clinical Stem Cell Center, Nanjing Drum Tower Hospital The Affiliated Hospital of Nanjing University Medical School Nanjing China

**Keywords:** gene modification, IL10, MSCs‐based therapy, periodontitis, the polarisation of macrophages

## Abstract

Regenerating the missing periodontium represents a difficult task during clinical management of periodontitis given that alveolar bone, periodontal ligament and cementum possess poor regeneration potential. The significant contribution of the inflammatory response to periodontitis occurrence and development has been extensively documented. Although the efficacy of treatments based on mesenchymal stromal cells (MSCs) has been proved, that of naïve MSCs treatment remains suboptimal. Herein, human MSCs stably expressing interleukin‐10 (IL‐10‐MSCs) were established and evaluated for their performance in treating ligature‐induced periodontitis rats. Utilising the electroporation method, a recombinant plasmid containing the human IL10 gene was introduced into human MSCs derived from umbilical cord to generate IL‐10‐MSCs. These IL‐10‐MSCs and blank vector transfected MSCs were transplanted into rat ligature‐induced periodontitis models by repeated injections via the periodontal and tail veins. Following MSCs transplantation, their therapeutic effects on the rat model and the underlying mechanisms were explored. IL‐10‐MSCs injections markedly promoted periodontal tissue regeneration, decreased bone resorption, and inhibited inflammation at the lesion area, relative to transplantation with unmodified MSCs as a control. Additionally, treatment with IL‐10‐MSCs respectively enhanced and decreased the proportions of macrophages undergoing alternative (M2) and classical (M1) activation at the lesion site. IL‐10‐MSCs exhibited more promising therapeutic efficacy against periodontitis in rats than naive MSCs and might represent a novel periodontitis treatment option.

**Abbreviations**
GBR: guided bone regeneration
GTR: guided tissue regeneration
PDLSCs: periodontal ligament stem cells
TRAP: tartrate‐resistant acid phosphatase
UC‐MSCs: human MSCs obtained from umbilical cord

## 1 Introduction

Periodontitis is a persistent non‐infectious condition accompanied by local inflammation. The formation of plaque biofilm is the major cause of the disease, which usually gradually destroys periodontal tissues involving cementum, alveolar bone, gingiva and periodontal ligaments, eventually resulting in tooth loss [1]. Plaque, in conjunction with host immune and environmental factors, promotes the development of periodontal inflammation over time. Therefore, regenerating the missing tissues is an even more important goal relative to restricting inflammation during periodontitis management [2]. Traditional treatments such as supragingival and subgingival scaling, along with antibiotics, often yield unsatisfactory outcomes. Periodontitis continues to progress even in patients who receive regular professional care. The nonsurgical periodontal therapies can prevent disease progression simply via physical removal of damaged tissues and pathogens, without facilitating comprehensive local tissue regeneration; surgical techniques with periodontal tissue‐regenerating effects, for example, Guided Tissue Regeneration (GTR), do not always yield desirable overall outcomes and seldom provide clinical predictability [3]. According to research, the real damage is caused by inflammatory mediators oversecreted by local immune cells rather than bacteria [4, 5]. Therefore, there have been many other approaches to controlling periodontitis recently, such as drug therapy, microbial therapy, stem cell therapy, and gene therapy [6]. At present, there are many studies on controlling periodontitis by regulating immune regulatory cells, especially regulatory T cells and regulatory B cells [7] or by regulating the differentiation direction of macrophages [8]. Similarly, stem cell therapy and gene therapy focus on mediating periodontitis‐related immune imbalances, promoting cell differentiation, promoting tissue repair, etc., to play a certain therapeutic effect [9]. However, periodontitis is related to many cells, cytokines, and even genes, which have not been fully studied.

Macrophages are a double‐edged sword in maintaining periodontal tissue homeostasis. On the one hand, macrophages can promote periodontal tissue repair by phagocytosing pathogens and producing anti‐inflammatory factors during periodontitis [10, 11]; on the other hand, however, they may be excessively generated from monocytes upon being stimulated by bacterial infection or bacterial secreted proteins (such as endotoxin) to overproduce pro‐inflammatory chemokines and cytokines, thereby further resulting in periodontal inflammatory reactions and bone tissue damage. After undergoing classical or alternative activation, macrophages can respectively polarise into the M1 or M2 phenotype. M1 macrophage activation may result in chronic inflammation and tissue damage [12, 13]; while the alternative activation phenotype facilitates the secretion of anti‐inflammatory cytokines including TGF‐β and IL‐10, which also possess angiogenic effects, to promote regeneration of tissues and wound repair [14, 15, 16]. Based on these findings, we hypothesised that enhancing the microenvironmental proportion and residing duration of M2 macrophages may benefit the repair of damaged periodontal tissues.

Interleukin‐10 (IL10) is an immunomodulatory cytokine that exerts its anti‐inflammatory effects by promoting macrophage M2 activation [17]. Many studies have identified IL‐10 as a key cytokine in periodontitis progression [18]. The excessive effect of IL10 on periodontitis recovery is possibly implicated with down‐regulation of proinflammatory cytokines and matrix metalloproteinases (MMPs) as well as osteoblastic activation [19]. Upon being infected with periodontal pathogens, IL‐10 levels can be detected in the saliva of patients with progressive periodontitis [20], and macrophages generate higher amounts of IL10, thereby inhibiting osteoclast formation and activating osteoblast differentiation [21]. Administering exosomes derived from M2 macrophages to a mouse periodontitis model has been shown to prevent pathological alveolar bone resorption and to increase IL‐10 mRNA expression [22]. Progranulin and IL‐37 can promote the high expression of IL‐10 in M2 macrophages, which can alleviate the progression of periodontitis in mice, suggesting that the remission of periodontitis is related to IL‐10 [23, 24]. Mouse ligation studies in a recently published paper have revealed that increased IL17 expression in IL10‐deficient mice stimulates macrophage differentiation in gingival tissue towards the M1 phenotype, resulting in alveolar bone loss [25]. Numerous studies have demonstrated that modulating IL‐10 expression—either directly or indirectly—improves outcomes in diseases such as colorectal cancer liver metastasis, liver fibrosis, and osteoarthritis [26, 27, 28]. Therefore, the intervention method of IL‐10 has a very important position in many immune‐related diseases. However, due to the IL10 protein's transient in vivo half‐life (generally < 1 h), its clinical application faces the major difficulty of maintaining a therapeutic drug concentration at the target site [29]. Consequently, few studies have reported successful disease treatment via direct IL‐10 protein injection.

Human MSCs obtained from umbilical cord have numerous advantages as a kind of practical seed cells for tissue regeneration, such as easy expansion and no ethical problems [30]. It is also easily genetically modified and is an excellent vector for transporting key target genes [31]. In patients with inflammatory periodontitis, umbilical cord‐derived MSCs transplantation could elevate bone regeneration and fresh connective tissue formation [32], but naïve MSCs treatment could not achieve its full clinical goal. Therefore, treatment strategies employing MSCs offer new hope in clinical management of periodontitis. Genetically modified MSCs are even more advantageous than their unmodified counterparts in terms of the efficacy and sustainability of targeted/effector molecule expression, thereby exhibiting superior therapeutic performance [33].

Herein, human MSCs obtained from umbilical cord (UC‐MSCs) were genetically modified (transfection through electroporation) to overexpress *IL10*, and highly‐expressing IL‐10 MSCs (IL‐10‐MSCs) clones were screened out. Then we thoroughly evaluated the general properties, differentiation ability, and immunomodulatory properties of IL‐10‐ MSCs. Afterwards, an in vivo investigation was carried out to appraise the performance of the genetically engineered cells in managing periodontitis. The findings reveal that, when compared to MSCs, IL‐10‐MSCs significantly promoted periodontal tissue regeneration, decreased alveolar bone resorption, and inhibited inflammation. We further found that IL‐10‐MSCs transplantation promoted macrophage M2 polarisation. Our research demonstrates that MSCs as target gene carriers are applicable for treating periodontitis through modulating the polarisation transition of M2/M1 macrophages.

## 2 Materials and Methods

### 2.1 Clinic‐Grade MSCs Isolation and Culturing

The protocols for experiments on human participants received approval from the institutional review board of Nanjing Drum Tower Hospital (approval number GCP‐SCP/17/2). Umbilical cord samples were processed into 2‐cm‐long fragments and flushed repetitively using DPBS (+Ca^2+^, +Mg2^+^, Hyclone, USA) containing 2% penicillin and streptomycin of CTS to flush out the arterial blood from the umbilical cord until it was blood‐free, after which the arterial veins were removed. Tissues were cut into 1 mm^3^ pieces and placed 1 cm apart in T75 culture flasks (Corning, USA) and inverted for 30 min in an incubator filled with 5% CO_2_ maintained at 37°C. The culture media containing α‐modified minimum essential medium (α‐MEM; Gibco, USA) plus 15% fetal bovine serum (FBS; Gibco, USA), 100 mg/mL streptomycin and 100 U/mL penicillin (Sigma‐Aldrich, USA) were slowly supplemented along the side wall of the flask and then placed in the incubator and replenished with fresh medium after 5 days. After about 10 days, the flask was gently tapped to dislodge the tissue mass, rinsed with PBS, the medium was changed, and the adherent cell morphology was examined. The culture medium was a half‐volume fluid refreshed once per 72 h. Upon reaching a confluency of 80%, the MSCs were passaged by trypsinisation (at a working concentration of 0.25%, Gibco, USA) of the attached cells. To avoid individual heterogeneity, we collected UC‐MSCs obtained from three second‐generation female infants as a pool to culture them together. UC‐MSCs at passage 3 (P3) were utilised for downstream analyses.

### 2.2 Preparation of IL‐10‐MSCs

#### 2.2.1 Gene Modification

The MSCs at P3 were used for electroporation to introduce human IL‐10 gene. The expression plasmids (HanBio) containing the human IL‐10 gene sequences under the control of the CMV promoter, which drives robust expression. The plasmid also includes a Kozak sequence for optimal translation, an SV40 poly(A) signal for mRNA stability, and a hygromycin resistance gene for selection of stable clones. Detailed plasmid construction and validation protocols have been described previously [31]. The plasmid was purified using an endotoxin‐free extraction kit (QIAGEN, Germany). We used a modified electroporation program of a CUY21EDIT II device (BEX Co. Ltd.) to perform 20 cycles of electroporation with the following parameters: drive voltage, 20 PdV; pulse voltage 150 PpV; pulse time, 10 ms. The mixture was inoculated in a 10‐cm dish after electroporation. MSCs were transfected with the plasmid blank vector serving as control. Cell status and mortality were assessed after 24 h of culture at 37°C and 5% CO_2_, after which hygromycin was added into medium to a concentration of 100 g/mL for selecting stably transfected clones for 10–12 days. Under the stereomicroscope (K‐400L, Motic China Group Co. Ltd.), individual colonies were manually picked, expanded, and screened by ELISA for IL‐10 secretion. Clone #18, exhibiting the highest IL‐10 output (≈2.8 ng/10^6^ cells/24 h), was selected for all downstream experiments [31].

#### 2.2.2 Identification and Culture of IL‐10‐MSCs

##### 2.2.2.1 Determination of Cell Value‐Added Ability

A Cell Counting Kit‐8 (CCK‐8; Beyotime, Shanghai, China) based on WST‐8 was employed for the cell proliferation assay. In 96‐well plates (4 × 10^3^ cells/100 μL per well), four equal aliquots of IL‐10‐MSCs and control MSCs suspensions were inoculated and respectively subjected to 1–7‐day‐long incubations. After that, each sample well was supplemented with 10 μL CCK‐8 solution and subjected to another 3‐h incubation at 37°C. The optical density values measured at 450 nm were determined with a plate reader. The experiments were repeated three times with the same cell source.

##### 2.2.2.2 Determination of Surface Marker Expressions

To investigate whether IL‐10 gene introduction affects the surface marker profiles of MSCs, 0.2 million cells were collected and subjected to a 30‐min incubation under darkness with phycoerythrin (PE) or fluorescein isothiocyanate (FITC)‐conjugated monoclonal antibodies for human CD34, CD45, CD90, CD19, CD44, CD105, CD73 and HLA‐DR (BD, USA). Three biological replicates were set up utilising cells from the same source. The cells were then rinsed thrice by 1× PBS and made into suspensions with washing buffer (BD FACSAria, USA) before flow cytometry assays. FlowJo V10 was utilised for flow cytometry data analysis.

##### 2.2.2.3 Determination of Osteogenic and Adipogenic Differentiation Ability

In DMEM (Gibco, USA) supplemented with 10% fetal bovine serum, ten‐thousand IL‐10‐MSCs and control MSCs were inoculated into each well of 6‐well plates. Upon reaching a confluency of 50%–70%, the cells were induced to undergo adipogenesis and osteogenesis with the corresponding differentiation media (Gibco). To assess the ability of adipogenesis and osteogenic differentiation, cells in the adipogenic group were subjected sequentially to formaldehyde (4%) fixation and Oil Red O staining (all reagents from Sigma‐Aldrich, USA), while cells in the osteogenic group were subjected sequentially to 95% ethanol fixation and Alizarin Red S staining (all reagents from Sigma‐Aldrich, USA).

##### 2.2.2.4 Establishment of Periodontitis Model

Vital River Laboratories (Beijing) provided the Wistar rats (*n* = 24) aged 12 weeks. To create a model of periodontitis of the second molars, anesthesia was performed on 18 animals using 10% chloral hydrate (Qingdao Yulong Algae Co. Ltd., China) at a dosage of 0.3 mL/100 g, and 4–0 non‐absorbable silk threads were ligated bilaterally under the gingiva of the maxillary second molars [34]. Six unmodeled rats served as the control group, and the model rats were divided into three groups at random including non‐treatment, MSCs treatment, and IL‐10‐MSCs treatment, with six rats in each group.

### 2.3 MSCs and IL‐10‐MSCs Therapy

Four weeks later, the silk threads were removed from all of the model rats, and the gingival buccal and palatal mucosa of the rats in both treatment groups were locally injected with MSCs (1 × 10^6^ cells/rat), IL‐10‐MSCs (1 × 10^6^ cells/rat) in 200 μL PBS/tooth [34]. A week later, the corresponding cells were injected into the tail veins of rats (5 × 10^6^ cells in 1000 μL PBS/rat) [35]. All rats were raised at the same temperature (21°C ± 2°C), humidity (55%), and a light/dark program of 12/12 h. Four weeks later, the animals were sacrificed by anaesthetic overdosing, and the maxilla was fixed with 4% paraformaldehyde.

### 2.4 MicroCT Imaging Re‐Establishment and Analysis

The rat maxilla was fixed inside a plastic tube, following which the crown and long maxillary axes were scanned in a MicroCT machine (SCANCOMEDICALAG, Switzerland) with the crown and long axes parallel to the scanning plane. The following parameters were adopted in the scans: 360° scanning, current 114 μA, voltage 70 kVp, 8 W, scanning thickness 17.5 μm, and high resolution. After scanning, the 3D image was re‐constructed. The proximal, middle, and distal points of the palatal and buccal sides were measured blindly between the cementoenamel junction and the alveolar bone crest (CEJ‐ABC distance) [36]. Each sample was analysed three times, with a one‐day interval between each measurement. Finally, each site's average value was calculated. Average values of the three points on each of the palatal and buccal sides and six points on both sides were calculated.

### 2.5 RAW264.7 Cell Culture

The RAW264.7 mouse macrophage cell line was maintained in DMEM (Gibco, USA) plus 10% inactivated FBS and penicillin–streptomycin at a concentration of 10,000 U/mL (15140122, Gibco). The growth media for MSCs and IL‐10‐MSCs were collected and tested for their activating effects on RAW264.7 cells. Briefly, 2 × 10^5^ RAW264.7 cells were seeded into each well of 12‐well plates. After culturing the cells for 24 h with DMEM/F12 containing IFN‐γ (R&D, USA) and LPS (Sigma‐Aldrich, USA) at concentrations of 2.5 and 500 ng/mL respectively, we collected the medium, designated as M1‐inducing medium, for further analyses. The medium used for culturing RAW264.7 cells without other reagents served as the negative control.

### 2.6 ELISA

RAW264.7 cells were incubated for 2 days with IL‐10‐MSC or MSC‐conditioned media supplemented with IFN‐γ and LPS. Afterwards, the media were harvested and subjected to centrifugation at 3000 rpm for 5–10 min, following which the supernatants were collected for ELISA. ELISA kits (EK206/3‐96 and EK282/4‐96, Multi Sciences, China) were used to detect TNF‐α and IL‐6 secretion levels from RAW264.7 for each group.

### 2.7 Histological and Immunostaining Assays

After fixing maxillary tissues inside 4% paraformaldehyde solution for at least 48 h, they were decalcified for 3 months with 5% EDTA (Gibco), paraffin‐embedded, and processed into 5‐mm‐thick sections (including gingiva, alveolar bone, buccal and palatal roots, and functions of maxillary second molars). For general histological examinations, we carried out tartrate‐resistant acid phosphatase (TRAP) and haematoxylin and eosin (H&E) staining analyses. Image Pro Plus 6.0 was employed for evaluating newly formed periodontal ligaments. The cell or tissue specimens were first immersed at 4°C overnight inside diluted polyclonal antibodies against arginase 1 (200‐fold dilution, 16001‐1‐AP), CD11B/integrin alpha M (100‐fold dilution, 66,519‐1Ig) and iNOS (200‐fold dilution, 18985‐1‐AP). All these primary antibodies were purchased from Proteintech Group Inc., China. Afterwards, the samples were subjected to a 1‐h incubation at ambient temperature inside 500‐fold‐diluted Alexa Fluor 488/568‐labelled secondary antibodies as appropriate (Invitrogen, USA). Lastly, the coverslips were mounted inside 4′‐6‐diamidino‐2‐phenylindole (DAPI) solution (ab104139, Abcam). The immunohistochemical assays were also performed for analysing protein levels of osteocalcin (OCN; 1:100, DF12303, Affinity, USA), TNF‐α (1:200, ab307164, Abcam), RUNX‐2 (1:200, ab236639, Abcam), and COX‐2 (1:200, ab283574, Abcam) in periodontal tissues around maxillary second molars.

### 2.8 Flow Cytometric Analysis

Briefly, 0.5 million RAW264.7 cells were used for inoculation in each well of six‐well plates. The conditions and procedures for cell culturing are the same as detailed above. After two days of culture, cells were subjected to incubation with F4/80‐FITC (11‐4801‐85, eBioscience, China) for half an hour at ambient temperature under darkness. After two PBS washes, cells sequentially underwent fixation, permeabilisation, and a 30‐min ambient‐temperature incubation with diluted antibodies targeting Arg1 and iNOS, which are respectively conjugated with eFluor 450 and PE fluorescent dyes. FACSAria (BD, NJ, USA) was used for measurements, and data were analysed with FlowJo Ver.10 (Tree Star, USA).

### 2.9 RNA Isolation and Relative Quantification of Gene Expression

The Invitrogen TRIzol reagent was employed for total RNA extraction from RAW264.7 cells. One microgram of the extracted RNA was then subjected to the synthesis of cDNA with HiScript II 1st Strand cDNA Synthesis Kit (Vazyme, China). The resultant cDNA was employed, in combination with gene‐specific primers and Vazyme ChamQ TMSYBR qPCR Master Mix, for quantitative real‐time PCR (qRT‐PCR). The reactions were carried out on the Quant Studio 6 Real‐Time PCR system (Thermo Fisher Scientific, USA). Utilising the 2^−△△CT^ algorithm, the relative abundance of target mRNAs was determined. Table 1 summarises the gene‐specific primers employed for qRT‐PCR assays.

**TABLE 1:** Sequences of qRT‐PCR primers.

Target gene	Forward primer (5′‐3′)	Reverse primer (5′‐3′)
iNOS	CCCTTCAATGGTTGGTACATGG	ACATTGATCTCCGTGACAGCC
Arg1	CTCCAAGCCAAAGTCCTTAGAG	GGAGCTGTCATTAGGGACATCA
CD11b	CCAAGACGATCTCAGCATCA	TTCTGGCTTGCTGAATCCTT
CD86	GGACATGGGCTCGTATGATT	TTAGGTTTCGGGTGACCTTG
CD206	TCTCCCGGAACCGACTCTTC	AACTGGTCCCCTAGTGTACGA

### 2.10 Statistical Analysis

Data obtained from three or more biologically independent replicates is summarised as mean ± standard deviation (SD). One‐way factorial analysis of variance (ANOVA) with Tukey's multiple comparison tests was employed for determining the statistical significance of intergroup differences, with a *p* value threshold of 0.05. All these analyses and result visualisation were performed with GraphPad Prism 9.0 (GraphPad Software, USA).

## 3 Results

### 3.1 Systematic Quality Evaluation of IL‐10‐MSCs

**FIGURE 1:**
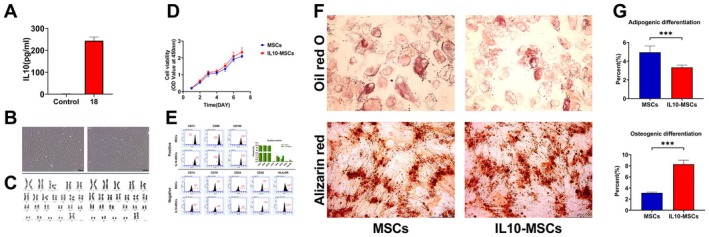
IL‐10‐MSCs quality evaluation results. (A) The abundance of IL10 in IL‐10‐MSCs clone (18#, P3–4) supernatant. MSCs transfected with a blank vector served as the control. (B) Microscopic observations confirmed that control MSCs (P6; left) and IL‐10‐MSCs (P6; right) exhibited similar morphology. (C) Representative findings corroborating that both control MSCs (left) and IL‐10‐MSCs (right) displayed normal karyotypes. (D) The CCK8 assay revealed no significant difference in proliferation between IL‐10‐MSCs (P8 generation) and control MSCs (P8 generation). (E) Flow cytometry assays revealed that IL‐10‐MSCs were dominated (> 95% of the total population) by CD73^+^/CD90^+^/CD105^+^ (mesenchymal markers) cells and contained minimal amounts (< 2% of the total population) of CD14^+^/CD19^+^/CD34^+^/CD45^+^/HLA‐DR^−^ (endothelial or haematopoietic markers) cells, satisfying the requirements for clinic‐grade MSCs. (F, G) IL‐10‐MSCs' capability of undergoing osteocytic and adipocytic differentiation as respectively evaluated through Alizarin Red and Oil Red O staining. IL‐10‐MSCs still had multi‐lineage differentiating potential, although their capacity for adipogenic and osteogenic differentiation was affected. *, **, ***, and **** respectively signify *p* values less than 0.05, 0.01, 0.001, and 0.0001 relative to the Ctrl group.

ELISA was first carried out to quantify IL10 secretion levels of screened positive MSCs (#18) and control MSCs, which revealed that #18 MSCs clone secreted significantly more IL‐10 compared with control MSCs clone (Figure 1A). For the #18 clone, IL10 gene modification had no effect on their long spindle morphology, proliferative capacity, and surface marker expression profile. As displayed in Figure 1B, unelectroporated naive MSCs (P6; left) and electroporated IL‐10‐MSCs (P6; right) exhibited similar morphology. Karyotype analysis was also performed on control MSCs and IL‐10‐MSCs to appraise the stability of their genetic materials. Both IL‐10‐MSCs (P6) and their control counterparts (P6) possessed normal karyotypes in terms of chromosome number (46, XX), size, length, morphology and mitotic positioning, and no abnormalities such as deletions, duplications, inversions, translocations, insertions, or ring chromosomes (Figure 1C). The cell proliferation rates were determined by CCK8 assays, which revealed comparable proliferation rates between IL‐10‐MSCs (P8) and control MSCs (P8) (Figure 1D). The expression profiles of cell surface protein markers were also compared between the two types of MSCs, revealing that both types were dominated (> 95% of the total population) by CD73^+^/CD90^+^/CD105^+^ cells and contained minimal amounts (< 2% of the total population) of CD14^+^/CD19^+^/CD34^+^/CD45^+^/HLA‐DR^−^ cells (Figure 1E).

Another critical concern is whether genetically modified MSCs retained their multispectral differentiation potential. To address this concern, we appraised the osteogenesis and lipogenic potential of IL‐10‐MSCs by carrying out Alizarin Red and Oil Red O staining assays. The results indicated that IL‐10‐MSCs retained a multispectral differentiation potential, but their lipogenic differentiation capacity was slightly down‐regulated and their osteogenic capacity was enhanced following IL‐10 modification relative to control MSCs (Figure 1F,G).

### 3.2 IL‐10‐MSCs Had Better Efficacy for Treating Rat Periodontitis

**FIGURE 2:**
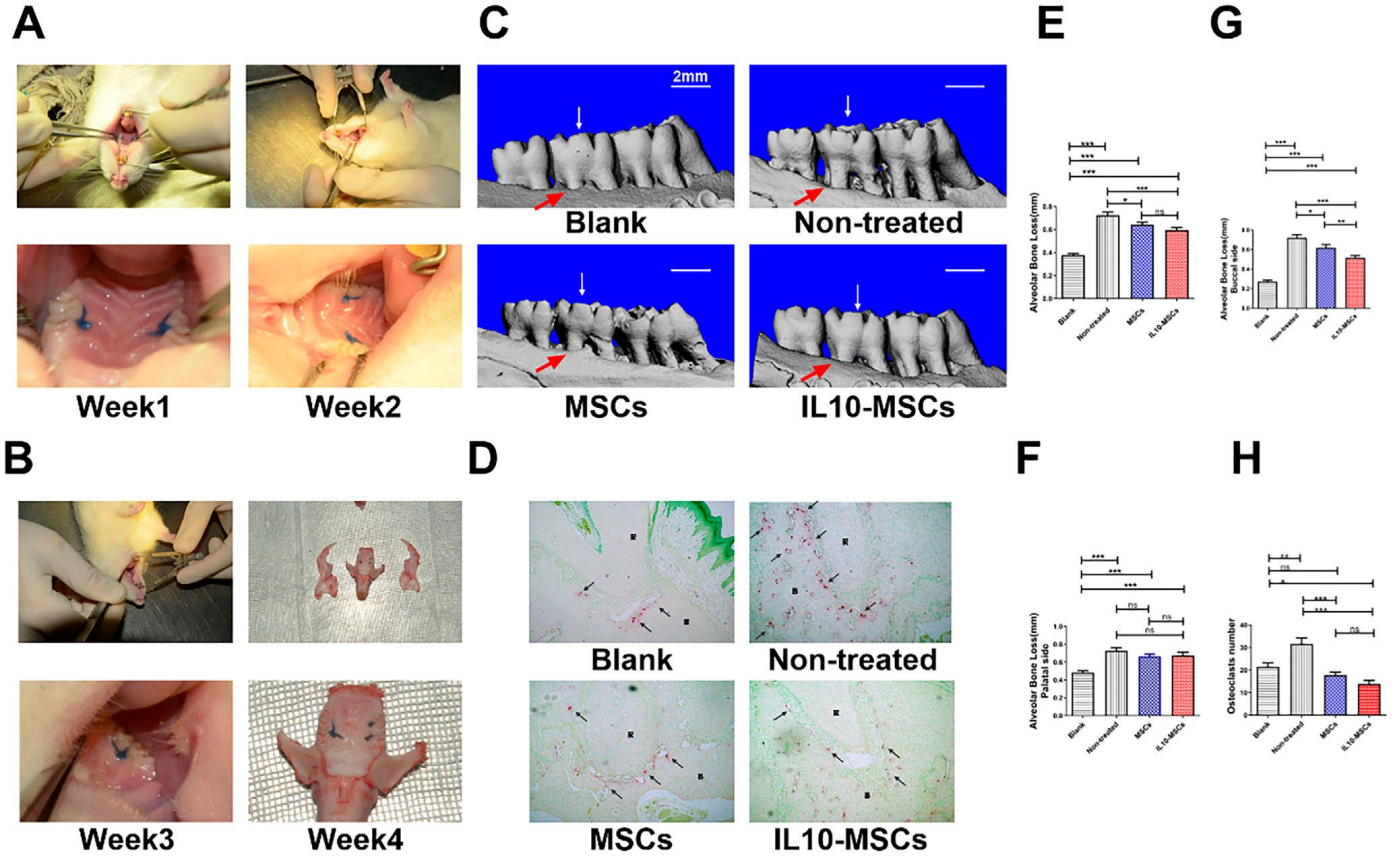
MSCs and IL‐10‐MSCs treatments suppressed the loss of bone tissues in periodontitis rats. (A, B) The successful establishment of the rat ligature‐induced periodontitis model. (C, E, G, F) The digitised images for the second molars of maxillae 3D‐reconstructed through Micro‐CT were quantified. (D, H) TRAP staining findings for the second molars of maxillae and quantification results of the number of osteoclasts in the corresponding periodontal tissues. *, **, ***, and **** respectively represent *p* values less than 0.05, 0.01, 0.001, and 0.0001 relative to the Ctrl group.

**FIGURE 3:**
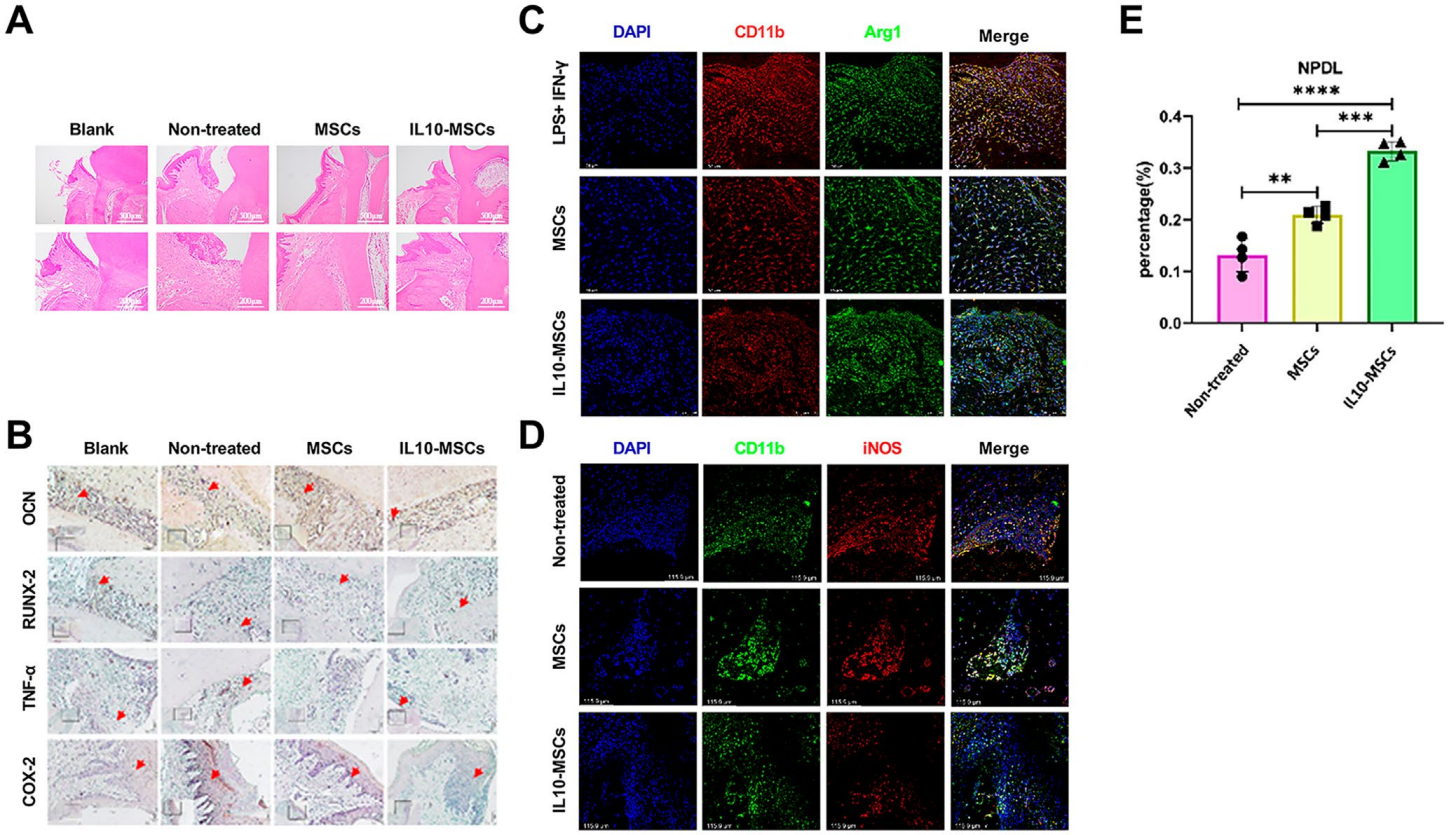
MSCs and IL‐10‐MSCs inhibited the fibre destruction and inflammatory response, and activated M2 macrophages in periodontitis rats. (A) H&E staining findings for the second molars of maxillae. (B) Immunohistochemical quantification of protein levels of OCN, RUNX‐2, TNF‐α, and COX‐2 in lesions of the second molars of maxillae. (C) IL‐10‐MSCs treatment evidently enhanced Arg‐1^+^/CD11b^+^ cell proportion ratio relative to those of the MSCs and vehicle groups. (D) IL‐10‐MSCs treatment markedly reduced iNOS^+^/CD11b^+^ cell proportion ratio relative to those of the other two groups. (E) Quantitation of the newly formed periodontal ligament among groups. **, ***, and **** respectively signify *p* values less than 0.01, 0.001, and 0.0001 relative to the Ctrl group.

Four weeks after filament ligation, the rat periodontitis models were successfully established (Figure 2A,B). microCT 3D reconstructions revealed significant second molar bone resorption in periodontitis rats relative to their sham‐operated counterparts. MSCs and IL‐10‐MSCs treatment could significantly decrease second molar bone resorption compared to the model group. The difference in alveolar bone height between the MSCs and IL‐10‐MSCs treatment groups did not reach a significant level (Figure 2C,E–G). The model rats had a significantly higher number of osteoclasts, while both the MSCs and IL‐10‐MSCs treatment groups exhibited markedly reduced osteoclast numbers in the alveolar bone, with no significant difference noted between these treatment groups (Figure 2D,H). In comparison with the sham‐operated rats, the model group presented with a disorganised thickening of the conjunctive epithelium, with some of the epithelium proliferating into the connective tissue in a striated or reticular pattern and containing more infiltrated immune cells. Notably, the gingival conditions of the IL‐10‐MSCs and sham groups were similar, with denser collagen fibers beneath the gingival sulcus (Figure 3A,E). The expressions of OCN, RUNX‐2, TNF‐α and COX‐2 were quantified through immunohistochemical assays and the findings (Figure 3B) indicated markedly enhanced TNF‐α and COX‐2 protein levels in the periodontal tissue with periodontitis in the model group, and the MSCs and IL‐10‐MSCs treatments were able to downregulate the levels of TNF‐α and COX‐2, inversely having more OCN and RUNX‐2 positive cells than the model group. Furthermore, IL‐10‐MSCs treatment resulted in the highest number of OCN and RUNX‐2 positive cells, indicating that IL‐10‐MSCs can indirectly facilitate the osteogenic differentiation of local endogenous cells or directly differentiate into osteogenic cells. Immunofluorescence showcased that the IL‐10‐MSCs group displayed a higher fluorescence intensity of Arg1 and CD11b double‐positive cells than the MSCs and model groups (Figure 3C), while having lower fluorescence intensity of iNOS positive population in the lesion site area relative to the other two groups (Figure 3D). These data demonstrated that IL‐10‐MSCs treatment significantly enhanced the M2 activation of macrophages at the lesion site when compared to control and MSCs treatment, implying this is one potential mechanism underlying the superior treatment effects of IL‐10‐MSCs against periodontitis.

### 3.3 IL‐10‐MSCs Modulate the M2/M1 Polarisation of RAW264.7 Cells Challenged With IFN‐γ^+^ LPS

**FIGURE 4:**
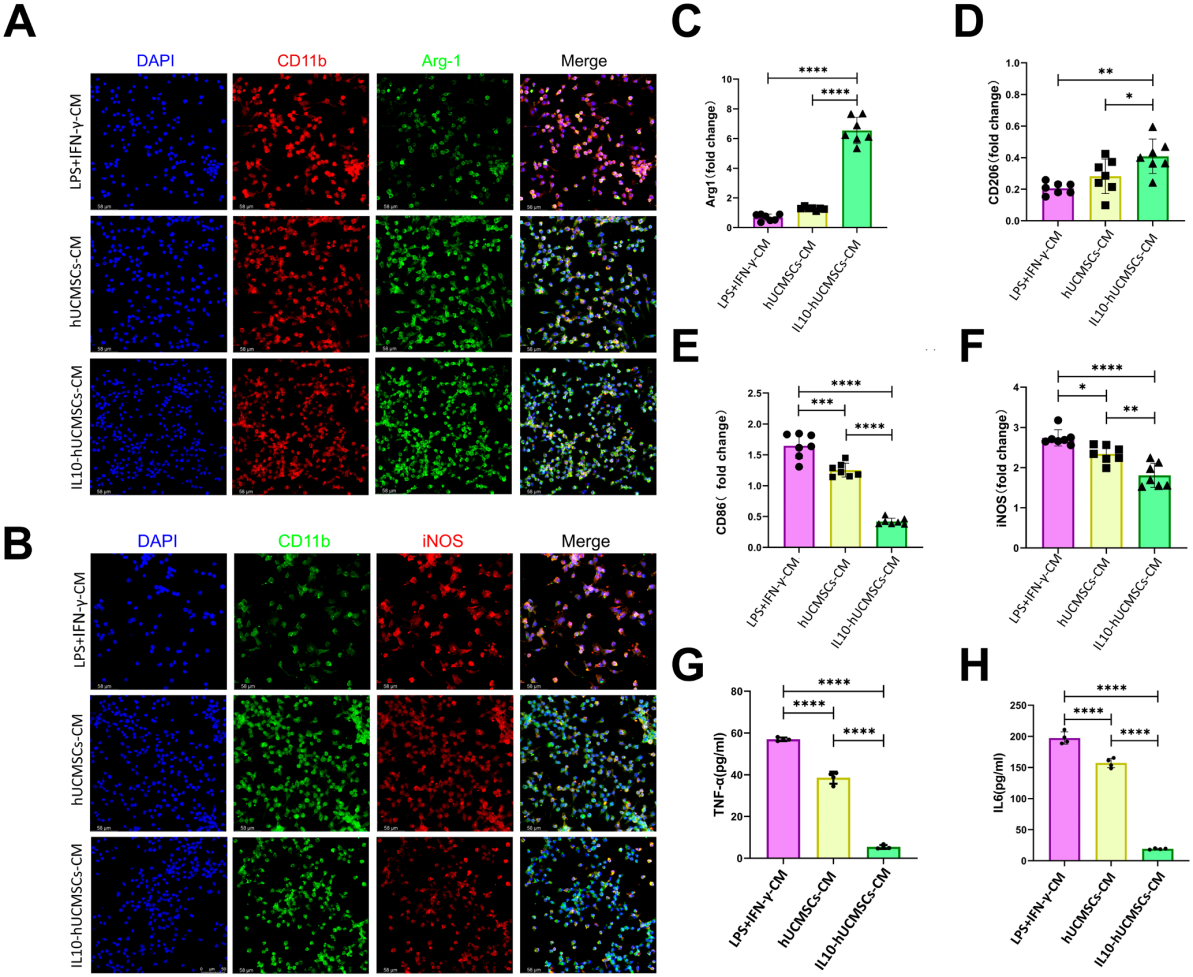
IL‐10‐MSCs facilitated M1 to M2 transition of RAW264.7 cells stimulated by IFN‐γ plus LPS. (A, B) Immunofluorescent analysis of iNOS and Arg1 protein levels in IFN‐γ^+^ LPS‐stimulated RAW264.7 cells treated with IL‐10‐MSCs‐CM or MSCs‐CM. (C–F) qPCR results signifying that IL‐10‐MSCs‐CM significantly enhanced the gene expression of M2 markers *CD206* and *Arg1* (C, D) while suppressed that of M1 markers *CD86* and *iNOS*. (E, F). (G, H) ELISA data reflecting that IL‐10‐MSCs‐CM obviously decreased IL‐6 and TNF‐α concentrations within cell culture medium.

**FIGURE 5:**
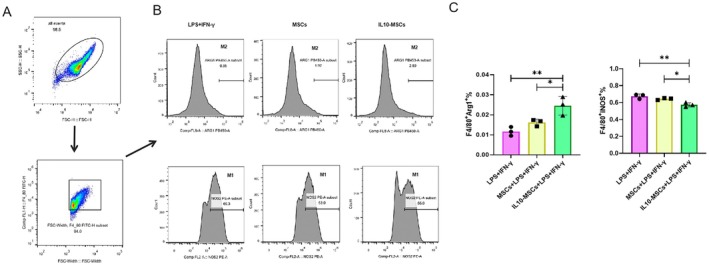
Flow cytometric data revealing the proportions of M1 (iNOS^+^/F4/80^+^) and M2 (Arg1^+^/F4/80^+^) RAW264.7 macrophages challenged by LPS plus IFN‐γ. A. Strategy of Flow cytometry, B and C represent the ratios of M1 (iNOS).

In periodontitis model, IL‐10‐MSCs treatment significantly increased the M2 phenotype of macrophages at the lesion site. Therefore, we investigated whether the IL10 secreted by IL‐10‐MSCs could facilitate macrophage M1 to M2 transition. Known regulators of the M1 phenotype of macrophages, LPS and IFN‐γ, were employed to stimulate mouse RAW264.7 macrophages into M1 macrophages. A total of 1 × 10^6^ IL10‐MSCs or MSCs were cultured for 2 days inside T75 flasks, after which the corresponding conditioned media (CM), namely IL‐10‐MSCs‐CM and MSCs‐CM, were collected for subsequent analyses. RAW264.7 cells were then cultured for 2 days within the aforementioned CM supplemented with IFN‐γ and LPS. Both MSCs‐CM and IL‐10‐MSCs‐CM significantly reduced RAW264.7 M1 polarisation while significantly enhanced its M2 polarisation, according to immunostaining (Figure 4A,B). Meanwhile, LPS plus IFN‐γ treated RAW264.7 exhibited a large number of stretched and lamellar filamentous pseudopods, resembling the classical phenotype of M1 macrophages. After IL‐10‐MSCs‐CM treatment, most cells reverted to rounded, M2‐polarised macrophages. To further quantify the M2/M1 transition, qPCR was used to detect the expressions of the corresponding markers. In comparison to the other two groups, IL‐10‐MSCs‐CM significantly enhanced the gene expressions of the M2 markers *CD206* and *Arg1* (Figure 4D,E) and reduced those of M1 markers *CD86* and *iNOS* (Figure 4F,G). ELISA experiments exhibited that IL‐10‐MSCs‐CM evidently reduced the protein expressions of M1 markers TNF‐α and IL6 relative to the control group (Figure 4H,I). These findings were further verified by flow cytometry data, which revealed that IL‐10‐MSCs‐CM co‐culture evidently elevated the proportion of F4/80^+^/Arg1^+^ RAW264.7 cells and reduced that of F4/80^+^/iNOS^+^ cells when stimulated by IFN‐γ plus LPS (Figure 5A–C).

## 4 Discussion

Periodontitis is a worldwide epidemic and a complex chronic inflammatory oral disease involving oral microflora, oral tissue, and immune cells [37, 38]. Periodontitis can damage periodontal tissue irreversibly, including the alveolar bone, periodontal ligament and cementum [39]. Disruption of the dynamic balance of oral microorganisms could enhance the risk of developing various systemic disorders such as myocardial infarction, colitis, and Alzheimer's disease [40, 41, 42, 43, 44]. Traditional periodontal therapy includes basic care, guided bone regeneration (GBR), and guided tissue regeneration (GTR), but the outcomes are patchy and their clinical predictability is subpar [45]. MSCs modulate the immune response via autocrine and paracrine mechanisms and then boost tissue regeneration through secreting a slew of protective bioactive factors rather than directly differentiating into target cells [46]. However, due to the limited therapeutic activity of immature cells, no MSC‐based treatment has been developed that can fully restore damaged periodontal tissue. As a result, creative cell‐based therapeutic approaches must be developed, including genetic modification and the optimisation of seed cell type, quantity, and delivery. Herein, the human IL10 gene was transfected into human MSCs to create an IL10 overexpressing stable MSC strain and then used for the first time in a rat periodontitis model to assess its therapeutic effect and investigate its mechanism. We discovered that IL‐10‐MSC treatment was more effective than control MSCs in terms of inhibiting alveolar bone resorption and reducing inflammatory cell infiltration. Furthermore, it was found that IL‐10‐MSC transplantation triggered the transformation of macrophage M2/M1 ratios at the lesion site. In vitro, IL‐10‐MSCs can also regulate RAW264.7 cell M2 polarisation challenged by LPS plus IFN‐γ. In conclusion, our findings demonstrate that treatment with IL10 gene modified MSCs is a more powerful cell therapy approach for periodontitis and has more benefits compared to control MSCs.

Research progress of single‐cell RNA sequences and spatial omics pointed out that the M1/M2 macrophage balance is the decisive switch between periodontal bone loss and regeneration [47, 48]. Although recombinant IL‐10 protein and IL‐10 coding vectors have been tested, their plasma half‐life of less than 1 h and rapid renal clearance have hindered their clinical translation [49]. Genetically engineered mesenchymal stem cells are becoming “living bioreactors”, maintaining the release of IL‐10 within the inflammatory niche. However, electroporated human umbilical cord mesenchymal stem cells have not yet been applied to ligation periodontitis models.

To adapt to the shorter half‐life of IL‐10, we combined periodontal pocket injection with subsequent tail vein infusion. Kanazawa et al. and Elhaieg et al. demonstrated that local administration of MSCs can achieve a 60% cell retention rate and better alveolar bone regeneration, while intravenous administration mainly exerts systemic immunomodulatory effects [50, 51]. Therefore, minimally invasive local injection should be given priority in future clinical translation, while systemic treatment approaches should be reserved for patients with extraoral inflammatory comorbidities.

MSCs are pluripotent progenitor cells with the capabilities of multi‐directional differentiation and self‐renewing. MSCs' immunomodulatory effect, combined with their multipotential, is extensively utilised in clinical investigation of various diseases, including tissue inflammation and autoimmune diseases [52]. MSCs are an appealing treatment option for periodontitis because they can target the inflammatory response while also promoting periodontal structure regeneration [53]. In addition to having high plasticity and developmental flexibility, human MSCs also have abundant sources, few ethical dilemmas, anti‐inflammatory capabilities, and the ability to regenerate [32, 54]. However, the naïve MSCs have limited therapeutic efficacy in a variety of diseases and could not reach our full goal. Thus, the naïve MSCs could be supposed to be the first‐regeneration therapeutic seeding cells. It is rational to establish next‐generation MSCs with enhanced therapeutic efficacy by targeting specific critical molecules according to the pathogenesis of certain diseases. IL‐10 is a famous wide‐spectrum anti‐inflammatory factor that possesses crucial protective effects in numerous diseases such as periodontitis. So, we established stable IL‐10 highly‐expressed MSCs strains to treat periodontitis. IL‐10 overexpression did not cause chromosome and surface marker profile alterations in MSCs including high expression levels of CD73, CD90, and CD105, and minimal expression levels of CD14, CD19, CD34, CD45, or HLA‐DR, but enhanced the potential of osteogenic and down‐regulated the capacity of lipogenic differentiation. IL‐10‐MSCs and MSCs groups expressed osteogenic differentiation markers like RUNX‐2 and OCN and inhibited osteoclast differentiation when compared to other groups. As a whole, IL‐10‐MSCs could remain the general traits of naïve MSCs but also affect some characteristics. In this study, the screened IL10‐MSCs up‐regulating the osteogenic differentiation potential perhaps is more helpful for bone‐defect related diseases. Evidently, IL‐10‐MSCs successfully restored the alveolar bone resorption and regenerated thick fibre cells at injured sites in a rat periodontitis model. IL‐10‐MSCs and MSCs treatments had more OCN and RUNX‐2 positive cells than the model group, and the IL‐10‐MSCs treatment induced the highest number of OCN and RUNX‐2 positive cells, indicating that IL‐10‐MSCs can facilitate the osteogenic differentiation of endogenous cells or directly differentiate into osteogenic cells.

The immune response is important in the development, progression, and regeneration of periodontitis. A promising method for treating periodontitis involves anti‐inflammatory strategies that use anti‐inflammatory cytokines. Recent research suggests that IL10 can prevent alveolar bone loss and periodontal inflammation while promoting bone formation [22, 25, 55]. However, the prolongation effect of IL10 protein in clinical trials is significantly restricted by its short half‐life (less than 1 h) in vivo and expensive price [56]. There is mounting evidence that combining MSCs and IL10 protein therapy can reduce inflammation in a variety of diseases, including experimental autoimmune encephalomyelitis [57], spinal cord injury [31] and ischemia–reperfusion injury [58]. In the present study, we treated the periodontitis using IL‐10‐MSCs to chronically release the IL10 in injured sites, leading to alleviating periodontal inflammation. Macrophages are essential innate immune cells that function as a double‐edged sword in periodontitis tissue damage and regeneration [59]. In a periodontitis animal model established by ligation [60], Viniegra et al. discovered that the M1 phenotype was actively metabolised in the early stages of inflammation, whereas the M2 phenotype proliferated during tissue healing. Yu et al. believed that periodontitis was associated with the enhancement of macrophage M1 and M2 phenotypes and that the transformation from the M2 to the M1 phenotype may be a key mechanism of periodontal tissue injury [61]. Drord et al. discovered that IL10 participated in the polarisation and function of M2 cells and had the ability to transform immature blood monocytes into M2 cells [62]. In this study, IL‐10‐MSCs transplantation increased the fluorescence intensity of M2 macrophage markers around the lesion while decreasing the fluorescence intensity of M1 macrophages. In vitro, IL‐10‐MSCs conditioned medium could promote the phenotypic transition of RAW264.7 cells, a mouse macrophage cell line, from M1 to M2. Thus, the transition of the M2/M1 ratio may explain one of the mechanisms by which IL‐10‐MSCs treatment inhibited periodontal tissue inflammation, and then promoted periodontal tissue regeneration, resulting in a better therapeutic efficacy compared with control MSCs.

There are some limitations needed to be further investigated in this manuscript. We did not specifically track MSC fate in tissues or quantify IL‐10 levels at the injury site. We speculate that MSCs may promote periodontal tissue repair and regeneration by regulating the local immune microenvironment by secreting factors such as IL‐10, rather than by differentiating into other cell types. In future research, we plan to use cell tracking technology to study the fate of MSCs in tissues. This will help us further clarify its mechanism of action and gain a more comprehensive understanding of the therapeutic effect of IL‐10‐MSCs in the treatment of periodontitis. Secondly, whether the IL‐10‐mediated increase in osteogenic differentiation potential directly offsets alveolar bone resorption in vivo remains to be determined. Additionally, periodontal ligament stem cells (PDLSCs) should be used as a control in future experiments to determine whether IL‐10‐MSCs can replace PDLSCs in terms of efficacy in the treatment of periodontitis.

## 5 Conclusions

**FIGURE 6:**
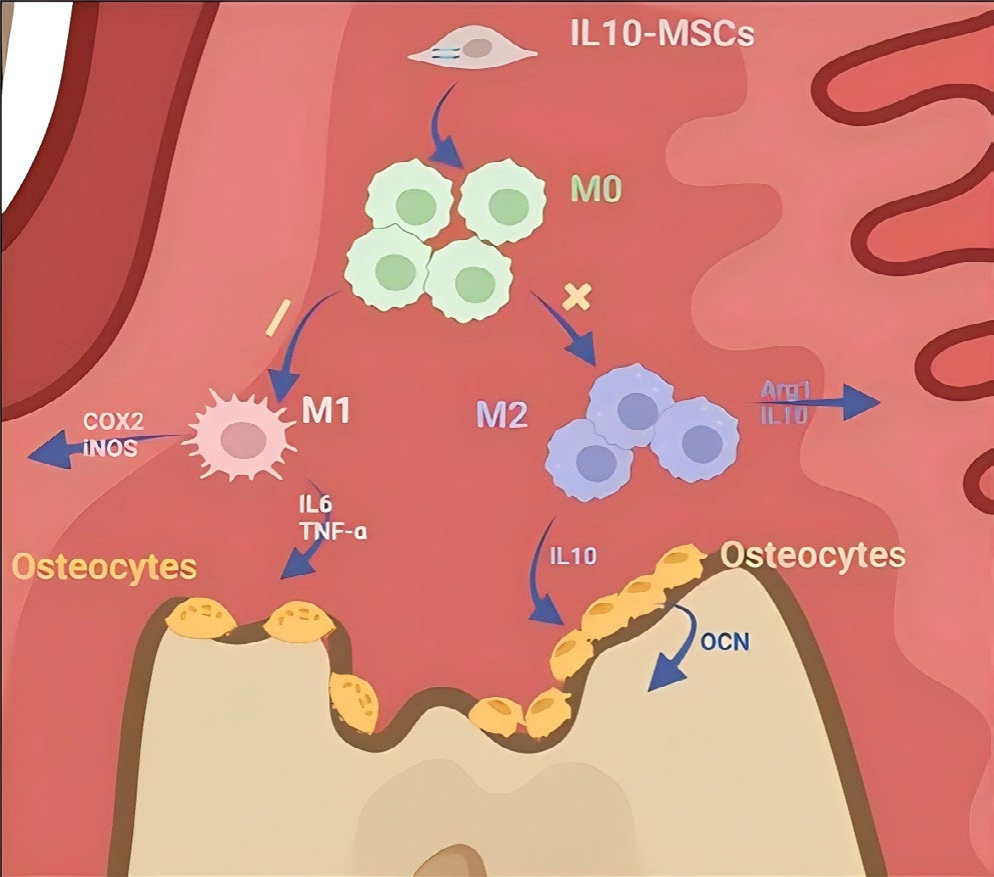
Proposed therapeutic strategy for periodontal disease using IL‐10‐MSCs.

After a thorough quality evaluation, we established overexpressed IL‐10‐MSCs and used them for the first time in a rat periodontitis repair model. IL‐10‐MSCs treatment has an evident better therapeutic efficacy via promoting M2/M1 macrophage switching at the injured site, including inhibiting inflammation and bone resorption, and promoting periodontal tissue regeneration (Figure 6). Our data demonstrate that genetically engineered MSCs overexpressing IL10 are suitable for the management of periodontitis and provide new insights into periodontitis cell therapy.

## Author Contributions

**Aerali Heinayati:** investigation (equal), writing – original draft (equal). **Xiaowei Jiang:** formal analysis (equal), methodology (equal), writing – review and editing (equal). **Tianyun Gao:** methodology (equal), resources (equal), writing – review and editing (equal). **Liudi Wang:** resources (equal), supervision (equal), validation (equal). **Bin Wang:** conceptualization (equal), funding acquisition (equal), project administration (equal). **Haiyan Qin:** conceptualization (equal), funding acquisition (equal).

## Ethics Statement

This research received approval from the Research Ethics Board of Nanjing Drum Tower Hospital [Title of approval project: Extracted Stem cells from clinical patient samples (tissue/blood/body fluid) and aborted fetal tissues for basic regenerative medicine and clinical disease treatment research; consent number: 2017‐161‐08, date: 2023‐11‐29]. Written informed consent was acquired from all participants, after which umbilical cord samples were obtained. All animal experiments were performed in the light of the Guide for the Care and Use of Laboratory Animals from the National Institutes of Health and this study was approved by the Institutional Animal Care and Use Committee of the Experimental Animal Center of Nanjing Drum Tower Hospital (approval No. 2019AE02030, date: 2019‐4‐27).

## Consent

The authors have nothing to report.

## Conflicts of Interest

The authors declare no conflicts of interest.

## Data Availability

The original data, materials and detailed experimental protocols associated with this research can be obtained from the corresponding author upon reasonable request.
